# Influence of ascorbic acid and α-tocopherol on the autoxidation and in vitro antifungal activity of amphotericin B

**DOI:** 10.18502/cmm.7.1.6178

**Published:** 2021-03

**Authors:** Mohammed Habib Belhachemi, Zahia Boucherit-Otmani, Kebir Boucherit, Sara Belmir

**Affiliations:** 1 Department of Biology, Faculty of Natural Sciences and Life Sciences and Earth and the Universe, University of Tlemcen, Algeria; 2 Department of Biology, Université de Ghardaia, Ghardaia, Algeria

**Keywords:** Amphotericin B, Antifungal activity, Ascorbic acid, α-tocopherol, Autoxidation

## Abstract

**Background and Purpose:**

Amphotericin B (AmB) is the standard treatment for systemic fungal infections; however, the formation of reactive oxygen species reduces the efficacy and stability of this molecule. The present study aimed to evaluate the effect of the combination of AmB with ascorbic acid and α-tocopherol on its autoxidation and antifungal activity.

**Materials and Methods:**

The antifungal activity against *Candida albicans* was evaluated by the viable cell counting method and checking their morphological changes with a scanning electron microscope. Monomer state of AmB was assessed by scanning the UV absorbance in the range of 300-450 nm and the lipid peroxidation was measured using quantification of thiobarbituric acid reactive-substances (TBARS).

**Results:**

Based on the findings, the addition of ascorbic acid (3×10^2^ µg/mL) and α-tocopherol (16 µg/mL) to the reaction medium of AmB increased its antifungal activity while maintaining its molecular stability. Moreover, the level of TBARS formed in the reaction medium of AmB was significantly reduced after combination with ascorbic acid and α-tocopherol.

**Conclusion:**

Given their availability, their anti-free radical activity, and their low toxicity, the incorporation of ascorbic acid and α-tocopherol into the reaction medium of AmB seems to be a promising approach to obtain an effective antifungal formulation.

## Introduction

The antifungal treatment has serious problems since there are no medications that are both effective and non-toxic; moreover, the increase in the resistance to antifungals can lead to treatment failure [ 1 - 3 ]. Among the most used antifungals, amphotericin B (AmB) is still the medication used against life-threatening systemic infections caused by various fungi, such as *Candida albicans* [ 4 , 5 ]. Despite the fact that AmB is a very active molecule, its use is limited due to problems of solubility, stability, and absorption which induce high toxicity [ 5 , 6 ]. This cytotoxicity is mainly due to the mechanism of action of AmB that is based on its interaction with membrane sterols and also to the production of reactive oxygen species [ 7 ].

Formation of these highly reactive intermediates would reduce the effective concentration of the medication in solution and also possibly contribute to its toxicity as has been reported for several unsaturated lipids [ 8 ]. However, the unstable nature of AmB probably reflects its susceptibility to autoxidative attack as suggested for members of the polyene group [ 9 - 11 ]. Nevertheless, the addition of an antioxidant to a yeast culture treated with AmB should improve its stability and the biological activity of the antibiotic for extended periods [ 10 , 11 ].

In this regard, the present study aimed to evaluate the effect of two antioxidant molecules, namely ascorbic acid and α-tocopherol, on the reaction medium of AmB in order to limit autoxidation reactions and increase its therapeutic efficacy.

## Materials and Methods

This study was carried out in the Antifungal Antibiotics Laboratory: Physical Chemistry, Synthesis, and Biological Activity, Department of Biology, University of Tlemcen, Tlemcen, Algeria (decree: N 256 du, 11/04/2001).

### Yeast strain and growth conditions

The yeast *C. albicans* ATCC10231 (American Type Culture Collection, Mendelevium, USA) coming from the Pasteur Institute of Paris. This stock was maintained by successive road repairs on Sabouraud dextrose agar (Sigma-Aldrich, Germany) and preserved at +4 °C. 

### Preparation of antifungal solutions

The pure AmB was obtained from the Bristol Myers Squibb laboratory in France. The stock solution was prepared by extemporaneously prepared antifungal in dimethyl sulfoxide (DMSO) (Sigma-Aldrich, Germany). From this stock solution, antifungal solutions were prepared at 20 µg/mL to measure the level of the anti-lipid peroxidation activity at 0.4 µg/mL and evaluate the antifungal activity [ 12 , 13 ]. The ascorbic acid and α-tocopherol (Sigma-Aldrich, Germany) were prepared extemporaneously in DMSO and added to the culture medium at time zero. 

### Antifungal susceptibility testing

The antifungal susceptibility testing of AmB alone and with ascorbic acid and α-tocopherol was performed according to the recommendations of the European Committee on Antimicrobial Susceptibility Testing (EUCAST) [ 14 ]. The minimum inhibitory concentration (MIC) values were determined in Roswell Park Memorial Institute 1640 medium (RPMI 1640) (Sigma-Aldrich, Germany) buffered with 3-(N-morpholino)-propane-sulfonic acid (pH 7.0 with 0.165 M) obtained from Sigma-Aldrich Chemie GmbH, Germany. 

Concentration of the *C. albicans* 10231 in the study was adjusted by measuring the absorbance in a spectrophotometer at a wavelength of 530 nm and adding sterile distilled water as required. A working suspension was prepared from a 1 in 10 dilution of the standardized suspension in sterile distilled water to yield 1-5×10^5^ colony-forming unit (CFU)/mL.The antifungal stock solution was two-fold diluted with RPMI of 16-0.03 µg/mL for AmB. A volume of 100 µL of inoculum suspension was added to each well except the sterility control. It must be mentioned that sterile water was added to the well instead. The microtitre plates were incubated for 24 h at 35 °C and the MIC was determined visually.

The MIC was defined as the lowest concentration of the antifungal agent that produced no visible fungal growth, compared to the medication-free control well. The control wells included the well without the antifungal (i.e. growth control), without microorganisms (i.e. sterility control), and with the solvent.

In order to determine the MIC of AmB and the concentrations of the combined vitamines, they were manipulated in several ways according to the EUCAST protocol with some modificationsto determine the effective concentrations against *C. albicans* strains. For this purpose, the range of concentrations of ascorbic acid (48×10^2^ to 9.37 μg/mL) and α-tocopherol (1.28×10^2^ to 0.25 µg/mL) was reduced while the concentrations of the antifungals were kept fixed [ 15 , 16 ]. At each manipulation, the concentration of AmB was reduced by a ratio of 1:2 until the achievement of the effective MIC of the antifungal which was compatible with one of the concentrations of the tested vitamins.

### Growth curves

Antifungal activity of AmB with and without vitamins against *C. albicans* was evaluated by time kill curve using the viable cell counting according to the methodology described by Klepser et al. [ 17 ] with some modifications. This broth-based method was prepared at the starting inoculum of *C. albicans* 10231 at 2×10^6^ CFU/mL by sampling the tubes or flasks that contained the control (i.e., the organism with no medication) and antifungal agent groups. Concentrations of the AmB alone or in combination with ascorbic acid and α-tocopherol were tested in accordance with the MIC results.

At predetermined time points (i.e., 0, 6, 12, 18, 24, and 33 h), the samples were continuously shaken and incubated at 30 °C. A 50 μL aliquot from each dilution was spread on a microscopic slide to determine the survivor colony count (i.e., CFU/mL). The kill curves were constructed by evaluation of the percentage growth inhibition at each time point in the presence and absence of the antifungal agent [ 18 ].

### Scanning Electron Microscopy

Morphological changes of *C. albicans* ATCC 10231 during growth by AmB alone and in combination with vitamins were observed by a TM-1000 scanning electron microscope (SEM) (Hitachi, Japan) with an accelerating voltage of 18 kV in the microscopy laboratory of the Physics Department at the University of Tlemcen. 

For purposes of the study, 10 mL of the *C. albicans* cell suspension at the starting concentration of 2×10^6^ CFU/mL was incubated at 30 °C for 33 h on a broth Sabouraud containing AmB with and without vitamins and the control group (i.e., the medium containing no AmB).

To perform the SEM analyses on samples, they were fixed in ethanol, stained with methylene blue, and rinsed in a buffer. Afterward, the smears were spread on the microscope slide and kept in the sterile Petri dishes [ 19 , 20 ]. The photomicrographs were processed and analyzed by Image J software (version 1.51a) to determine the charge and cell dimensions of *C. albicans* ATCC 10231 during the stationary phase in the absence and presence of antifungal agents [ 19 ].

### UV–visible absorbance

The medication concentrations in dispersions were calculated based on absorbance at 406 nm after appropriate dilution in methanol. Monomer state of AmB was determined by scanning the UV absorbance of AmB within the range of 300-450 nm using the Specord 200 PLUS spectrophotometer (Analytik Jena, Germany) [ 21 ].

For evaluation of the monomeric state of AmB formulations (with and without vitamins) at 37 °C, the samples were dissolved in phosphate buffer (pH 7.4/10 mM) and the spectroscopic measurements were carried out at 10^-5^ M of AmB concentration after dilution of each AmB formulation with absolute methanol. Measurements were repeated in triplicate over a period of 1, 18, 24, and 33 h, and the samples were stored in a place protected from light at room temperature during the experiment [ 21 , 22 ].

### Lipid peroxidation assay

Lipid peroxidation activity was determined according to the method of Sakanaka S. and Tachibana Y. with some modifications [ 23 ]. Moreover, the formations of TBARS were determined by mixing 1 mL of the egg yolk solution and 0.5 mL of AmB alone (20 µg/mL) or in combination with vitamins (the concentrations were according to the MIC results). Subsequently, 2 mL of FeSO4 at 0.01 mM was added to the prepared solution.

The mixture was shaken at 37 °C for 15 min; afterward, 1 mL of 2.5% trichloroacetic acid was added to it. The mixture was mixed well and centrifuged at 4000 ×g for 20 min. In addition, 3 mL of the supernatant was mixed with 2 mL of 0.8% 2-thiobarbituric acid and heated to 100 °C for 10 min. Absorbance of the mixture was measured at 532 nm in triplicate.

To evaluate the results of this experiment, a standard curve was prepared with malonyldialdehyde (MDA) from 1,1,3,3-tetramethoxypropane (Sigma-Aldrich, Germany).

### Statistical analysis

Statistical analysis of the different experimental groups was performed using XLSTAT software (Version 2014.5.03). Moreover, the significance of differences in the antifungal activity and the monomeric state of AmB formulations was determined by the Mann–Whitney U test. It must be noted that a p-value of less than 0.05 was considered statistically significant.

## Results

### Antifungal susceptibility testing

To determine the MIC of AmB in combination with ascorbic acid and α-tocopherol, several microdilution tests were carried out by decreasing the MICs of the antifungal. According to the obtained results, a significant decrease (from 0.5 to 0.12 µg/mL) was observed in the MIC of AmB in combination with vitamins against *C. albicans* ATCC 10231. However, the concentrations compatible with ascorbic acid and α-tocopherol with the obtained MIC were 3×10^2^ µg/mL and 16 µg/mL, respectively. 

### Growth curves

**Figure 1:**
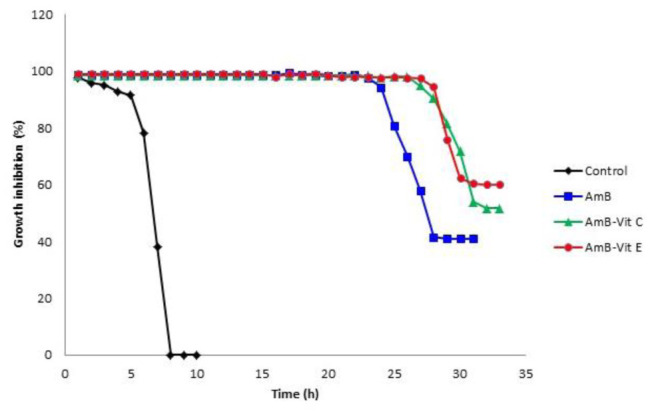
Effect of the addition of ascorbic acid (Vit C=3×10^2^ µg/mL) and α tocopherol (Vit E=16 µg/mL) on the antifungal activity of amphotericin B (AmB=0.4 µg/mL) against *Candida albicans* ATCC 10231. The data are presented with mean values and standard errors of fungal counts. Each point represents an average of triplicate measurements (P=0.0368, AmB vs. AmB-Vit C; P=0.0237, AmB vs. AmB-Vit E; P=0.0589, AmB-Vit C vs. AmB-Vit E; Mann-Whitney U- test).

Antifungal activity of AmB with and without ascorbic acid and α-tocopherol against *C. albicans* ATCC 10231 is represented by the growth curves shown in Figure 1. According to the results, the addition of ascorbic acid (3×10^2^ µg/mL) and α-tocopherol (16 µg/mL) to the reaction medium of AmB (0.4 µg/mL) increased the efficacy of the antifungal agent to 11% and 19%, respectively. Percentage of growth inhibition of fungal cells was estimated at 41% in the presence of AmB alone with a maximum growth of 59×10^6^ CFU/mL and a latency phase of 24 h.

On the other hand, it was noted that the addition of ascorbic acid and α-tocopherol to the reaction medium of AmB increased its antifungal activity against the strain of *C. albicans* ATCC 10231 with a percentage inhibition of 52% and 60%, respectively, which represents cell loads of 48×106 CFU/mL and 40×106 CFU/mL, respectively. This inhibition in growth is accompanied by a prolongation of the latency phase which goes from 24 h (AmB alone) to 27 h (AmB/Ascorbic acid) and 28 h (AmB/α-tocopherol).

### Scanning electron microscopy

**Figure 2:**
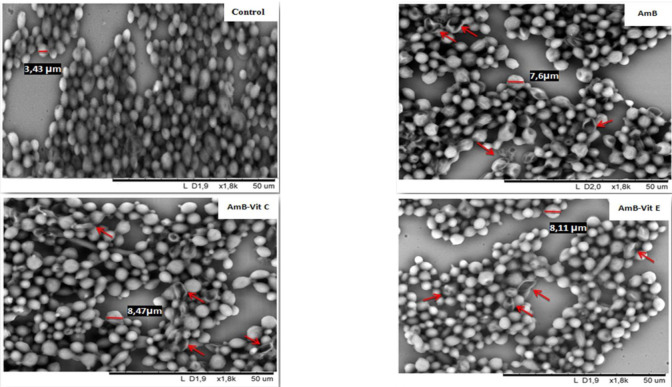
Results of scanning electron microscopy of *Candida albicans* ATCC 10231 cultured in the absence and presence of the antifungal agents during the stationary phase (amphotericin B [AmB]=0.4 µg/mL, ascorbic acid [Vit C]=3×10^2^ µg/mL, α tocopherol [Vit E]=16 µg/mL).

Figure 2 shows the morphological changes of *C. albicans* ATCC 10231 cultured in the absence and presence of the antifungal agents during the stationary phase using a SEM. There was an increase in the cell sizes of yeasts incubated in the presence of AmB alone (0.4 µg/mL) and in combination with ascorbic acid (AmB-Vit C; 3×10^2^μg/mL) and α-tocopherol (AmB-Vit E; 16 µg/mL) after 33 h (which corresponds the stationary phase). Moreover, it must be noted that there was a burst in the *C. albicans* cells incubated in the presence of AmB alone. 

Based on the results of the Image J software (version 1.51a), the mean values of the cell sizes of *C. albicans* ATCC 10231 were 7.60, 8.47, and 8.11 μm in the presence of AmB alone and AmB in combination with ascorbic acid and α-tocopherol, respectively. it is noteworthy that the same value was 3.43 μm in the control group.

### UV–visible absorbance

**Figure 3:**
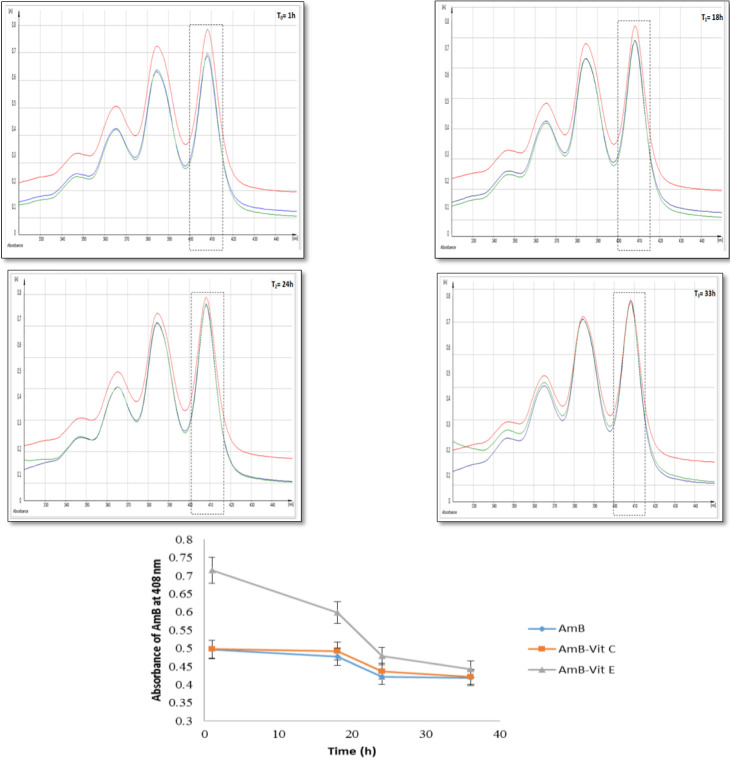
Characteristic spectra of the monomeric state of amphotericin B (AmB=10^-5^ M) in the presence of ascorbic acid (Vit C=3×10^2^ µg/mL) and α tocopherol (Vit E=16 µg/mL) as a function of time. Each point represents the mean of triplicate measurements (P=0.4189, AmB vs. AmB-Vit C; P=0.0002, AmB vs. AmB-Vit E; P<0.0001, AmB-Vit C vs. AmB-Vit E; Mann-Whitney U- test).

Monomeric state of AmB was evaluated by measuring the UV-visible absorbance of them and the results are shown in Figure 3. Spectra of AmB with

and without ascorbic acid and α-tocopherol displayed strong absorption in the 408 nm region with different intensities. Indeed, AmB keeps its molecular stability where the characteristic peak always retains its maximum value at 408 nm.

In contrast, a decrease was noted in the absorbance intensity of AmB over time. Spectral analysis of AmB (10^-5^ M) in combination with ascorbic acid (3×10^2^ µg/mL) and α-tocopherol (16 µg/mL) revealed a hyperchromic effect during 24 h in the combination of AmB and α-tocopherol.

### Lipid peroxidation assay

**Figure 4:**
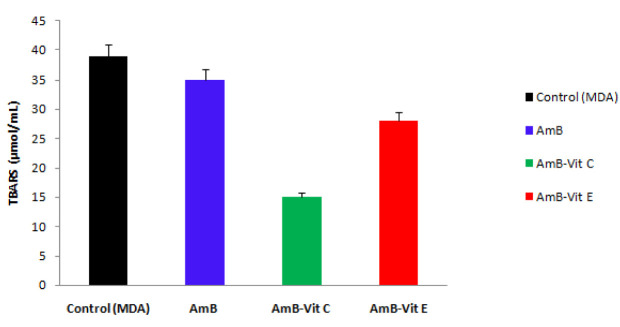
Concentration of thiobarbituric acid reactive substance (TBARS) in the reaction medium of amphotericin B in the absence and presence of ascorbic acid (Vit C=3×10^2^ µg/mL) and α tocopherol (Vit E=16 µg/mL), compared to malonaldehyde (MDA=20 µg/mL). Data represent the mean±SD values (n=3).

Figure 4 represents the concentration of malonaldehyde in the reaction medium of AmB (20µg/mL) in the absence and presence of ascorbic acid (3.10^2^µg/mL) and α-tocopherol (16 µg/mL) as a function of the measured absorbance values.

Based on the obtained results, AmB had a great capacity to produce malonic dialdehyde in the reaction medium with a concentration of 35 μM. This concentration was significantly reduced by the addition of ascorbic acid and α-tocopherol in the reaction medium of AmB to 15 μM and 28 μM, respectively.

## Discussion

A preliminary study based on data from the literature [ 11 , 16 , 24 , 25 ] has indicated that the addition of ascorbic acid and α-tocopherol to the reaction medium of AmB reduces its toxicity to human red blood cells without affecting its antifungal activity [ 10 ]. In this regard, the present study aimed to determine the effective concentrations of vitamins which are compatible with the AmB molecule in order to limit the autoxidation reactions and increase its therapeutic effectiveness. These two molecules were selected due to their chemical nature, anti-radical activities, and low toxicities.

The MIC of AmB and concentrations of the combined vitamins were determined according to the EUCAST protocol. Based on the results, there was a decrease in the MIC of AmB from 0.5 to 0.12 µg/mL against *C. albicans* ATCC 10231 in combination with ascorbic acid and α-tocopherol with final concentrations of 3×10^2^ μg/mLand 16 µg/mL, respectively. This result showed an apparent synergistic AmB-antioxidant relationship according to Beggs WH [ 26 ].

Based on the results of the growth curves, the addition of ascorbic acid and α-tocopherol with final concentrations of 3×10^2^ µg/mL and 16 µg/mL, respectively, to the reaction medium of AmB (0.4 µg/mL; corresponds to the MIC against *C. albicans* ATCC 10231 [ 13 ]) inhibited the growth of *C. albicans* ATCC 10231. Moreover, the latency phase was extended from 24 h in the presence of AmB alone to 28 h in its combination with the vitamins. 

This result is in line with those of the studies performed by Atmaca and Çiçek [ 25 ], as well as Baran and Thomas [ 27 ] which have indicated that the addition of antioxidant molecules to AmB stabilizes this molecule against auto-degradation and prolongs its antifungal activity against a strain of yeast *C. albicans*.

In addition, the microscopic study (i.e., SEM) revealed that the incorporation of ascorbic acid (3×10^2^ µg/mL) and α-tocopherol (16 µg/mL) to the reaction medium of AmB (0.4 µg/mL) reduces the cell load and increases the size of *C. albicans* ATCC 10231 cells, compared to the yeasts incubated in the presence of AmB alone [ 19 ]. Investigation of the effect of antioxidants on the reaction medium of AmB offers a simple way to solve the problem of therapeutic efficacy and stability of this antifungal molecule. Therefore, the addition of ascorbic acid and α-tocopherol to the reaction medium of AmB did not influence the molecular stability and value of the peak characteristic of the monomer state of AmB which remained constant at 408 nm.

According to Biémont [ 28 ], the study of the spectra of a large number of molecules has made it possible to establish correlations between structures and absorption maxima [ 29 ]. However, the hypochromic and hyperchromic effects caused a variation in the intensity of the maximum peak over time. Indeed, this variation can suggest the size of the AmB molecule and the capacity to form a molecular complex with ascorbic acid and α-tocopherol.

Monomeric state of AmB is the most effective with less toxicity due to its solubility in the aqueous medium. According to Nielsen et al. [ 16 ], the addition of α-tocopherol to mixed micellar solutions increases their solubilization which may explain the better efficiency and stability of the combination of AmB and α-tocopherol [ 30 ]. 

According to Gaboriau et al. [ 31 ], AmB promotes the formation of reactive oxygen species, such as hydroperoxides, conjugated dienes, and certain aldehydes. Formation of these molecules is the result of the oxidation reaction of lipids that are added to the primary and secondary products [ 32 , 33 ]. According to Eymard and Genot [ 34 ], in the case of unstable products, such as hydroperoxides and conjugated dienes (i.e., primary products), these measurements do not allow the determination of the exact level of lipid oxidation. The reason is that these intermediate products are quickly broken down into secondary products. The main stable aldehyde formed is MDA which is recognized as a biomarker of lipid peroxidation.

Addition of the antioxidant molecules to the AmB reaction medium appears to be an effective approach to limit the spread of the lipid oxidation reaction and stabilize the AmB molecule in order to increase its activity against *C. albicans*. Results of this study are consistent with those of a study performed by Kovacic and Cooksy [ 35 ] who concluded that ascorbic acid and α-tocopherol form a stable and active complex with AmB to increase their antifungal activity.

## Conclusion

Addition of ascorbic acid and α-tocopherol to the reactive medium of AmB could provide an answer to the solubility, stability, and toxicity problems commonly encountered in chemotherapy. These advantages are due to the antioxidant power of the added molecules; they protect AmB against oxidative damage and retain its molecular stability.
